# Assessment of a commercially available automatic deformable registration system

**DOI:** 10.1120/jacmp.v11i3.3175

**Published:** 2010-06-09

**Authors:** B. Gino Fallone, D. Ryan C. Rivest, Terence A. Riauka, Albert D. Murtha

**Affiliations:** ^1^ Department of Physics University of Alberta Edmonton Alberta Canada; ^2^ Department of Oncology University of Alberta Edmonton Alberta Canada; ^3^ Department of Medical Physics Cross Cancer Institute Edmonton Alberta Canada; ^4^ Department of Radiation Oncology Cross Cancer Institute Edmonton Alberta Canada

**Keywords:** image‐guided radiation therapy, deformable image registration, commercial registration systems, registration validation

## Abstract

In recent years, a number of approaches have been applied to the problem of deformable registration validation. However, the challenge of assessing a commercial deformable registration system – in particular, an automatic registration system in which the deformable transformation is not readily accessible – has not been addressed. Using a collection of novel and established methods, we have developed a comprehensive, four‐component protocol for the validation of automatic deformable image registration systems over a range of IGRT applications. The protocol, which was applied to the Reveal‐MVS system, initially consists of a phantom study for determination of the system's general tendencies, while relative comparison of different registration settings is achieved through postregistration similarity measure evaluation. Synthetic transformations and contour‐based metrics are used for absolute verification of the system's intra‐modality and inter‐modality capabilities, respectively. Results suggest that the commercial system is more apt to account for global deformations than local variations when performing deformable image registration. Although the protocol was used to assess the capabilities of the Reveal‐MVS system, it can readily be applied to other commercial systems. The protocol is by no means static or definitive, and can be further expanded to investigate other potential deformable registration applications.

PACS numbers: 87.19.xj, 87.56.Da, 87.57.nj

## I. INTRODUCTION

In recent years, deformable image registration has become a very important component in a number of image‐guided radiation therapy (IGRT) and adaptive radiation therapy (ART) protocols. It has been applied to problems such as autosegmentation,^(^ ^1^ ^,^ ^2^ ^)^ four‐dimensional (4D) treatment optimization,^(^ ^3^ ^,^ ^4^ ^,^ ^5^ ^,^ ^6^ ^,^ ^7^ ^)^ dose accumulation^(^ ^1^ ^,^ ^8^ ^)^ and tumor growth/regression analysis.^(^ ^9^ ^)^ Varied objectives in these and other potential applications, compounded by the fact that often the lack of a gold standard makes true assessment impossible,^(^ ^10^ ^)^ make validation of deformable image registration algorithms very difficult.

A number of approaches have been applied to the problem of deformable registration validation. Although inherently limited, visual assessment has been used in combination with other methods to qualitatively validate registration results.^(^ ^11^ ^,^ ^12^ ^,^ ^13^ ^,^ ^14^ ^)^ It has become common practice to compare algorithms by evaluating similarity measures such as the sum of square intensity differences (SSD), correlation coefficient (CC), and mutual information (MI) upon completion of image registration.^(^ ^13^ ^,^ ^15^ ^,^ ^16^ ^,^ ^17^ ^)^ It is assumed that there is a direct correlation between enhanced similarity values and registration accuracy. Although these methods may be sufficient for relative comparison, they provide little information on the absolute accuracy of registration.

Various deformable thoracic^(^ ^18^ ^,^ ^19^ ^,^ ^20^ ^)^ and pelvic^(^ ^14^ ^,^ ^21^ ^,^ ^22^ ^)^ phantoms with varying levels of design complexity have been manufacturing and applied to deformable registration validation. Xiong et al.^(^ ^23^ ^)^ used inflatable balloons with radio‐opaque markers to assess their algorithm for bladder deformation in CT images acquired prior to high dose rate (HDR) vaginal cuff brachytherapy. A gel‐balloon phantom containing plastic beads molded to the gel that propagate based on levels of balloon inflation was used by Lu et al.^(^ ^15^ ^)^ to validate their free‐form deformable registration program. Although practical, authors have argued that phantoms have limited value in validating deformable registration algorithms because they cannot fully assess the impact of anatomical variations on the algorithm's performance.^(^ ^24^ ^,^ ^25^ ^)^

A useful quantitative method of assessing registration is to apply a known simulated mathematical transformation to a patient image, generating a synthetic warped image. Registration of the original and warped images allows for direct comparison of the true displacement field with that generated by registration. Lu et al.^(^ ^15^ ^)^ applied a harmonic function to a 2D pelvic CT image, while Lau et al.^(^ ^26^ ^)^ warped 2D $T_{1}$‐weighted MR brain images with five types of synthetic mathematical transformations. Intuitively, the closer the simulated transformation resembles clinical anatomical variations, the more relevant this method is for validation. As such, this procedure is enhanced by using a second independent deformable registration algorithm to generate the known transformation for validation of the algorithm in question. Fiducial based thin‐plate spline^(^ ^27^ ^)^ registration has been used to warp thoracic CT^(^ ^18^ ^)^ and abdominal CT^(^ ^14^ ^)^ images for synthetic transformation based deformable image registration assessment. The major downside of using simulated warped images for registration validation is that the method is not applicable for registration of images of differing modalities.

The most commonly employed and perhaps the most accurate method of quantitatively validating deformable registration is to compare the position of anatomical landmarks or the overlap of regions of interest (ROI) in registered images. Measuring distances between manually identified points such as vascular and bronchial bifurcations is a common approach to validating thoracic CT deformable registration.^(^ ^3^ ^,^ ^4^ ^,^ ^5^ ^,^ ^6^ ^,^ ^7^ ^)^ Brock et al.^(^ ^28^ ^)^ also identified landmarks on thoracic and abdominal MR images when validating a finite element model (FEM)‐based deformable registration system. Various measures of volume overlap evaluated using radiation oncologist delineated prostate contours have been used for validation of deformable registration of $T_{2}$‐weighted pelvic images with and without inflated endorectal coils.^(^ ^29^ ^,^ ^30^ ^,^ ^31^ ^)^ Volumetric‐based methods have also been used when validating deformable registration of pelvic CT^(^ ^1^ ^,^ ^2^ ^)^ and head and neck CT^(^ ^16^ ^)^ images.

The majority of the aforementioned references share a commonality in that the authors develop a novel algorithm that requires some procedure to assess its capabilities. However, the problem of assessing a commercial black box deformable registration system prior to clinical implementation has not been addressed – in particular, an automatic registration system in which the deformable transformation is not readily accessible. Proper quality assurance and acceptance testing of such systems is necessary to identify subtle limitations for various clinical applications. Using a collection of novel and established methods, we have developed a comprehensive protocol for the validation of automatic deformable image registration systems over a range of IGRT applications. This work predominantly focuses on anatomical images in which one‐to‐one correspondence is desired between registered images. Deformable registration involving functional images will be addressed briefly. The protocol has been applied to the Reveal‐MVS Fusion Workstation (Mirada Solutions, Ltd.).

## II. MATERIALS AND METHODS

As is the case for other technological advancements implemented in radiation therapy, the extent of the capabilities and limitations of a commercial system must be understood prior to its use in any IGRT deformable registration application. Given the wide range of anatomical variations across the population, different imaging modalities and scanning protocols and the number of potential applications of deformable registration in IGRT, a single standardized algorithm is probably insufficient. As such, an automatic commercial deformable registration system would require multiple algorithms or, at the very least, multiple registration settings in order for it to be relevant over a spectrum of applications. With this in mind, our protocol consists of four separate components: a phantom study to determine the system's general tendencies, relative validation of different registration settings, absolute verification of the system's intra‐modality and inter‐modality deformable registration tools. All patient images were retrospectively incorporated into this study with local research ethics board consent. The terms source and target will be used to describe registrations throughout this paper, using the standard notation that the source image is registered to the target image. In all deformable registrations on the Reveal‐MVS system, images were initially aligned using the system's automatic rigid registration tools. All analysis was performed using 3D image sets.

### A. Registration software

The Reveal‐MVS system has automatic rigid and deformable registration capabilities, as well as a separate PET/CT scanner automatic motion correction option specifically designed to account for the typically minor anatomical variations present between PET and CT scans on a combined or hybrid PET/CT system. The deformable registration algorithm is proprietary, thus severely limiting the authors' knowledge of its actions. However, the roots of the company Mirada Solutions Ltd. (currently Mirada Medical, Oxford, UK) can be traced to the Wolfson Medical Vision Laboratory at the University of Oxford.^(^ ^32^ ^,^ ^33^ ^)^ The automatic deformable registration and PET/CT motion correction tools have user defined settings for stiffness (None, Soft, Medium, Stiff), speed (Slow, Medium, Fast), and refinement (Coarse, Medium, Fine), giving rise to 36 permutations of setting combinations. Although the methods are not open source, some information about the settings is provided with the software's documentation. If stiffness is set to ‘None’, no constraints are set to the registration, whereas a stiffness of ‘Stiff’ typically limits displacements to less than 0.5 cm. ‘Soft’ and ‘Medium’ settings typically limit displacements to 5 cm and 2 cm, respectively. The speed setting sets the approximate registration time to less than a minute, 3–4 minutes or in excess of 10 minutes, for ‘Fast’, ‘Medium’ and ‘Slow’ settings, respectively. The refinement setting determines the level of localization at which the deformations are applied to images. For example, the ‘Coarse’ setting results in deformations applied at a resolution of approximately 5 cm. Resolutions of 2 cm and 0.5 cm are typical of the ‘Medium’ and ‘Fine’ settings, respectively. The deformation function generated after registration of the source and target images is not accessible, although it can be saved and subsequently loaded and reapplied to the original source or any other image. The system allows for export of deformed images, features built‐in contouring tools with exporting capabilities, but is unable to import ROI contours delineated on other systems. All import and export functions are based on the DICOM standard.

### B. Phantom study

A number of authors have argued that phantoms have limited value in quantitatively evaluating deformable registration software because they cannot fully assess the impact of anatomical variations on the algorithm's performance.^(^ ^24^ ^,^ ^25^ ^)^ In principle, we agree with this assertion. However, it was believed that a simple, site nonspecific, multi‐modality phantom may yield pertinent information about an unknown deformable registration algorithm. As such, we included a phantom study in our protocol not for quantitative evaluation of Reveal‐MVS, but as a qualitative assessment tool that was designed and implemented with two objectives. First, to determine if the system has any general tendencies that might influence the registration of patient images; and second, to determine whether or not the system's actions when registering images were modality dependent.

**Figure 1:**
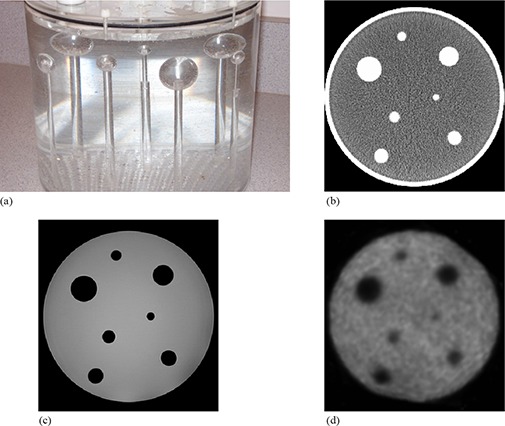
Side view photographic image of the phantom (a) showing the seven solid spheres attached to plastic rods that are screwed into the phantom base; sample axial CT (b), MRI (c) and PET slices (d) of the target image setup.

We began with a commercial cylindrical water phantom and manufactured a circular water equivalent plastic slab with a 12 × 12 grid of quarter‐inch threaded holes 8 mm apart and attached the slab to the inside base of the phantom. Seven solid plastic spheres of varying diameters attached to quarter‐inch diameter rods were inserted in the base so as to be fairly evenly distributed throughout the phantom. Reference target CT (512 × 512×62 voxels; $0.9\times 0.9\times 2.0\;{\text{mm}}^{3}$), $T_{1}$‐weighted MRI (512 × 512×21 voxels; $0.6\times 0.6\times 5.0\;{\text{mm}}^{3}$) and PET (144×144×30 voxels; $4.0\times 4.0\times 4.0\;{\text{mm}}^{3}$) images (Fig. 1) were acquired on the clinical imaging systems used for radiation therapy patients in our clinic at the time the phantom study was performed. CT images were acquired on a Picker PQ 5000 (Marconi Medical Systems, Inc., Cleveland, OH) scanner, while a Gyroscan Intera (Philips Medical Systems, Best, The Netherlands) was used for MRI image acquisition. An 82 MBq FDG solution was injected into the water phantom prior to imaging on an Allegro (Philips Medical Systems) PET scanner. System resolutions for the CT, MRI and PET imaging protocols used to acquire the phantom images are approximately 0.7 mm, 0.8 mm and 6.5 mm, respectively. The size and location of individual plastic spheres was modified in various combinations of replacements or 8 mm translations. Source images of each of the different phantom setups were acquired with all three imaging modalities. Since we were predominantly interested in the system's treatment of the modified spheres, the plastic rods were digitally removed from all images using the method described by Crouch et al.^(^ ^22^ ^)^ in removing brachytherapy seeds from pelvic CT images. Intra‐modality deformable registration of each source image to its respective target image was performed on the Reveal‐MVS system. PET and MRI source images were also registered to the target CT image for inter‐modality investigation. Analysis was limited to a qualitative visual inspection of the deformable registration results.

### C. Relative validation

The objective of the second component of the protocol is to determine the optimal deformable registration settings for a collection of IGRT applications. It should be re‐iterated that patient images were acquired retrospectively and, unlike in the phantom study, the authors could not dictate which imaging systems were used. As such, different scanners were used for different applications; however, there were no variations amongst each individual application. The following five image combinations, selected based on availability and the desire to include multiple anatomical sites and imaging modalities, were investigated.

***PET/CT**:* Studies have demonstrated that staging detection with FDG PET leads to improved patient management and often impacts radiation therapy planning in non‐small cell lung carcinoma (NSCLC) patients.^(^ ^34^ ^,^ ^35^ ^)^ Combining functional PET and anatomical CT information presents many challenges, but a number of them have been addressed with the advent of combined PET/CT scanners.^(^ ^36^ ^)^ In a single imaging study, these systems consecutively acquire CT and PET images with the patient in a fixed position on a single imaging couch in a timeframe on the order of minutes. The design does not eliminate, but significantly reduces the level of anatomical variations between patient PET and CT images acquired on independent systems. PET (144×144×178 (median) voxels; $4.0\times 4.0\times 4.0\;{\text{mm}}^{3}$) and CT (512×512×151 (median) voxels; $1.2\times 1.2\times 5.0\;{\text{mm}}^{3}$) images were acquired on a Gemini PET/CT unit (Philips Medical Systems). System resolutions are approximately 0.7 mm and 6.5 mm for the CT and PET components of the Gemini unit, respectively.

***Longitudinal PET**:* Longitudinal or temporal imaging studies where a patient is successively imaged to monitor change in a disease state, or to assess the effectiveness of treatment is a common practice in radiation therapy. We address this particular application by registering post‐treatment follow‐up images to pre‐treatment baseline images (144×144×213 (median) voxels; $4.0\times 4.0\times 4.0\;{\text{mm}}^{3}$) acquired on an Allegro PET scanner, which has a measured system resolution of 6.5 mm.

***Longitudinal thoracic CT**:* The utilization of deformable image registration in 4D radiotherapy of the lung has been well documented.^(^ ^3^ ^,^ ^4^ ^,^ ^5^ ^,^ ^6^ ^,^ ^7^ ^)^ Registration of thoracic CT images acquired during different respiratory phases allows for modeling of respiratory motion and improved delineation of target margins. For simplicity we register longitudinal thoracic CT (512×512×43 (median) voxels; $0.8\times 0.8\times 7.5\;{\text{mm}}^{3}$) studies imaged on a diagnostic quad‐slice Mx8000 scanner (Philips Medical Systems), which has a measured resolution of approximately 0.7 mm.

***Pelvic MRI to planning CT**:* It has been cited that the positions of both the prostate apex and base are often misidentified on pelvic CT images, and that rigid registration of MRI and CT can improve target delineation during prostate treatment planning.^(^ ^37^ ^)^ However, if the prostate has translated or has become slightly deformed with respect to surrounding anatomy between imaging studies, rigid registration may not result in accurate overlap of the MRI and CT prostate volumes. Deformable registration has the potential to further improve target delineation by accounting for internal prostate motion. $T_{1}$‐weighted MRI (512 × 512×35 (median) voxels; $0.8\times 0.8\times 6.0\;{\text{mm}}^{3}$) and planning CT (256×256×91 (median) voxels; $1.9\times 1.9\times 3.0\;{\text{mm}}^{3}$) images were acquired on an Intera 3T (Philips Medical Systems) magnet and a PQ 5000 CT scanner, respectively. The system resolution for the protocol used for MRI image acquisition is approximately 2.2 mm, while the corresponding value for the CT images is 0.7 mm.

***Pelvic megavoltage CT (MVCT) to planning CT**:* The protocol for patients treated on the Hi·Art II (TomoTherapy, Inc., Madison, WI) helical tomotherapy unit at our clinic calls for the acquisition of daily megavoltage CT (MVCT) (512×512×29 (median) voxels; $0.8\times 0.8\times 6.0\;{\text{mm}}^{3}$) images with the patient in treatment position. Pre‐treatment MVCT images are beneficial in that they provide daily image guidance^(^ ^38^ ^)^ and allow for calculation of the daily delivered dose.^(^ ^39^ ^)^ It has been demonstrated that accurate deformable registration of daily MVCT and planning CT images permits the evaluation of accumulated dose distributions delivered over the coarse of treatment.^(^ ^9^ ^)^ Planning CT (256×256×91 (median) voxels; $1.9\times 1.9\times 3.0\;{\text{mm}}^{3}$) images were acquired on a PQ 5000 system. Approximate resolutions for the MVCT and planning CT acquisition systems are 1.7 mm and 0.7 mm, respectively.

Five image pairs for each individual application were imported into the Reveal‐MVS system for this study. After initial automatic normalized mutual information based rigid alignment, every source image was automatically registered to its respective target image using each of the possible 36 automatic deformable registration settings. The automatic motion correction tool was used for PET/CT registration in place of automatic deformable registration. Deformed images were exported and postregistration similarity measures were evaluated using in‐house software developed in C++. Symmetric correlation ratio, mutual information, and correlation coefficient values were calculated for each intra‐modality image pair, with the latter excluded from inter‐modality analysis. A simple qualitative visual assessment of fused images was used to evaluate the generally accepted relationship that similarity measure optimization corresponds to improved registration accuracy.

### D. Intra‐modality registration

Two of the registration applications evaluated in the previous section, one intra‐modality and the other inter‐modality, were selected for analysis of absolute registration accuracy. For the intra‐modality application, mathematically simulated deformations were used for absolute validation of longitudinal thoracic CT deformable registration. Unedited and synthetically deformed patient images that differed by a known transformation $T_{0}$ were registered using Reveal‐MVS. Absolute comparison of the true transformation $T_{0}$ and that produced by the commercial system *T* was achieved by calculation of the displacement error,^(^ ^26^ ^)^ which is defined as

(1) $$DE=\frac{1}{\sqrt{N}}\sqrt{\sum_{i=0}^{N-1}\varepsilon_{i}^{2}}$$

where *N* is the number of image voxels and for each voxel *i*, $E_{i}$ is the voxel error, the displacement between the true position of each deformed voxel and the position obtained by registration. In addition to full 3D analysis, axial components of DE values were also evaluated.

In order to best represent clinical reality, transformations were selected by registering baseline to follow‐up patient images with a standard B‐spline parameterized free form deformable (FFD) registration algorithm implemented in the Insight Segmentation and Registration Toolkit (ITK). ITK is an open‐source, object‐oriented software package that consists of a collection of C++ classes designed for image processing, segmentation and registration that can be implemented in user‐developed software. The registration need not be perfect so long as the generated transformation and the synthetic deformed baseline image were clinically relevant and plausible. Synthetic images were then registered to the original baseline images on the Reveal‐MVS system using the optimal settings determined by relative evaluation.

Upon conducting this procedure on each of the five image pairs analyzed in the previous section, we recognized that additional information could be extracted from the process in order to further enhance analysis of the commercial system's absolute capabilities. FFD registration is an iterative process and not only can the final iteration be considered a clinically relevant transformation, each preceding iteration can be viewed as representing clinical plausibility. As such, B‐spline transformation parameters for nine additional iterations from each FFD registration were used to produce synthetic images. In all five FFD registrations, convergence required greater than ten iterations, so additional iterations were selected to obtain B‐spline transformations representing varying degrees of deformation.

**Figure 2:**
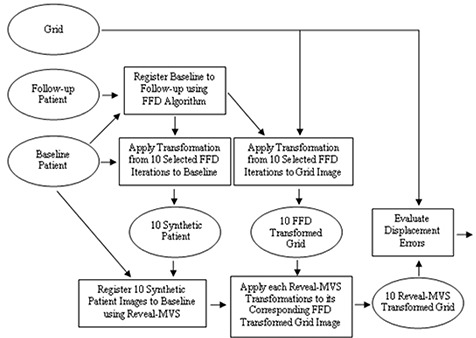
Flowchart showing the steps and images involved in the synthetic transformation based intra‐modality validation study. Rectangles represent steps or actions, while images are denoted by ellipses. Patient baseline, follow‐up and grid images are procedural inputs and evaluated displacement errors are outputs.

Calculation of the displacement error for Reveal‐MVS registrations was hindered by the fact that transformations in Reveal‐MVS cannot be accessed by the user. To overcome this problem, we developed a grid‐based system in which baseline patient images were modified by initially setting the intensity of each to zero. A set of randomly selected individual image voxels was then modified to have non‐zero intensity values creating a nonuniform grid image. All image modification was performed with a MATLAB (The MathWorks, Natick, MA) script. Grid images were then deformed using the same known B‐spline transformation $T_{0}$ applied to the original baseline image and deformed images were subsequently imported into Reveal‐MVS. The Reveal‐MVS registration derived transformation *T* was applied to the deformed grid image, which was then exported for analysis. Perfect registration on Reveal‐MVS would result in the exported image being equivalent to the original nondeformed nonuniform grid image and a displacement error value of nil. Both grid images were imported into analysis software developed in C++ and the approximate displacement error was calculated using the locations of each of the grid points. A flowchart is used to summarize the intra‐modality registration validation procedure in Fig. 2.

Recognizing that the accuracy of our proposed DE evaluation method was limited by a number of factors including statistics, and the potential merging or crossover of grid points, we felt that validation of its efficacy was required. A collection of known B‐spline transformations defining varying magnitudes of deformation was applied to a randomly selected grid image. B‐spline functions were obtained for all iterations during the registration of the selected longitudinal thoracic CT images with our FFD registration program based on ITK. After setting the intensity of all image voxels in the baseline image to zero, either 500, 1000, 3000, or 5000 randomly selected voxels were set to non‐zero values. The deformed and original grids were imported into our evaluation software for calculation of approximate DE values, which were compared with true displacement error values extracted from the known B‐spline functions. The process was repeated using differing quantities of random grid points to determine the optimal number of non‐zero intensity voxels to employ.

### E. Inter‐modality registration

Finally, we evaluated the Reveal‐MVS system's ability to deform pelvic MRI images to planning CT images through analysis of radiation oncologist delineated prostate contours. However, an important factor must be considered when performing volumetric analysis of prostate contours drawn and CT and MRI images. It has been demonstrated through independent studies that contoured prostate volumes are greater in CT than in MRI images.^(^ ^37^ ^,^ ^40^ ^)^ Kagawa et al.^(^ ^37^ ^)^ observed that CT contours often erroneously included sections of seminal vesicles, the base of the bladder, adjacent structures such as venous plexus and fibromuscular stroma, neurovascular bundles, and the anterior rectal wall. In fact, inter‐modality variation has been demonstrated to exceed inter‐observer variation^(^ ^40^ ^)^ and its consequences should influence the procedural design of this study.

Each patient MRI image was registered to its respective planning CT using the optimal deformable registration setting determined through relative validation. Prostate volumes were contoured on each of the five CT and five unregistered MRI pelvic images by a single radiation oncologist on Reveal‐MVS. Each patient's unregistered MRI image was displayed on a monitor adjacent to the registration system in an attempt to improve prostate CT delineation. Contours were exported and a C++ program was used to convert patient images into binary images in which voxels corresponding to prostate had intensity of one, while all other voxel intensities were set to zero. Binary MRI images were imported into Reveal‐MVS and appropriate transformations were applied, generating deformed binary MRI images for comparison with each patient's binary CT image.

Volumetric analysis of binary CT and deformed binary MRI images consisted of evaluation of the mean surface distance between contours and the two most common measures of region overlap,^(^ ^25^ ^)^ the Tannimoto Coefficient (TC) and Dice Similarity Coefficient (DSC). Given a CT contour volume $V_{\mathrm{CT}}$ and a MRI contour volume $V_{\mathrm{MRI}}$, TC and DSC are defined by

(2) $$TC=\frac{V_{CT}\cap V_{MRI}}{V_{CT}\;\cup V_{MRI}}$$

(3) $$DSC=\frac{2(V_{CT}\cap V_{MRI})}{V_{CT}+V_{MRI}}$$

**Figure 3:**
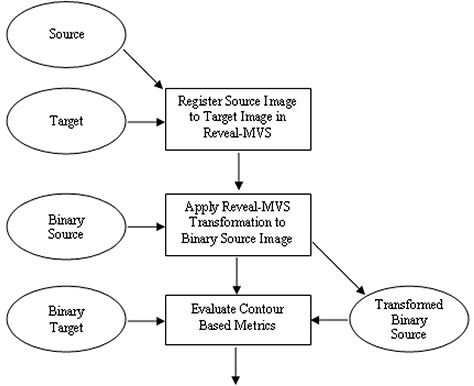
Flowchart showing the steps and images involved in the contour based inter‐modality validation study. Rectangles represent steps or actions, while images are denoted by ellipses. Source, target, binary source and binary target images are procedural inputs, and evaluated contour based metrics (Tannimoto Coefficient, Dice Similarity Coefficient and mean surface distance) are outputs.

Both measures have possible values ranging from 0 for no overlap to 1 for perfect agreement between volumes. A flowchart is used to summarize the inter‐modality registration validation procedure in Fig. 3. In addition, all four protocol components are summarized in Table 1.

**Table 1:** Summary of the validation protocol procedure, including the analysis performed and images evaluated for each of the protocol's four components.

	*Analysis*	*Images Registered*
Phantom study	Qualitative visual analysis of registration	CT to CT; MRI to MRI; PET to PET; MRI to CT; PET to CT
Relative validation	Comparison of post‐registration similarity measures	PET/CT; Longitudinal PET; Longitudinal thoracic CT; Male pelvic MRI to planning CT; Male pelvic MVCT to planning CT
Intra‐modality	Evaluation of post‐registration axial and 3D displacement errors (DE)	Synthetic thoracic CT to original thoracic CT
Inter‐modality	Evaluation of post‐registration contour metrics	Male pelvic MRI to planning CT

## III. RESULTS

### A. Phantom study

**Figure 4:**
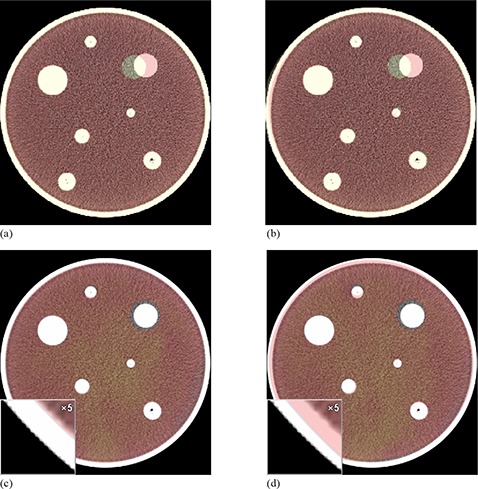
Axial slices of colored source images overlaid on greyscale target phantom images for two selected registrations: rigid alignment of CT images (a) in which a single target has been translated 8 mm; CT images after deformable registration on Reveal‐MVS (b) demonstrating that the system does not move the sphere back to its original location; rigid alignment of MRI and CT images (c) in which a sphere in the MRI has approximately doubled in volume. An inset window with 5x magnification depicts the differences between the phantom perimeter in the MRI and CT images resulting from a lack of MRI signal from the plastic phantom perimeter. MRI and CT images (d) after deformable registration in which the system attempts to deform the MRI phantom volume to that of the CT phantom volume while ignoring the volumetric differences in the modified target.

A very consistent trend was observed in all of the phantom image deformable registrations on the Reveal‐MVS system regardless of phantom modification or image modality. Whether one of the plastic spheres was translated or replaced with a different sized sphere, the registration software performs little or no image warping in the vicinity of the modified sphere. This is illustrated in Fig. 4 for a CT to CT registration in which a sphere was translated 8 mm and a MRI to CT registration where a sphere was approximately doubled in volume. The illustration for the second example contains an additional inset window in which a portion of the outside edge of the phantom is magnified with respect to the remainder of the phantom. Due to a lack of signal from the plastic phantom perimeter, the phantom diameter is smaller in the MRI image than in CT and, as a result, the system attempts to deform the MRI phantom volume to that of the CT phantom volume. These and other observations suggest that the commercial system is more apt to account for global deformations than local variations when performing deformable image registration.

### B. Relative validation

**Figure 5:**
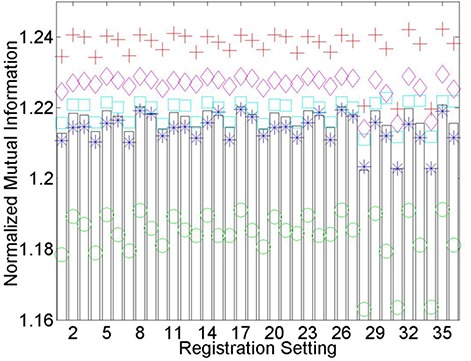
Longitudinal thoracic CT postdeformable registration normalized mutual information (NMI) values for each of the 36 registration setting combinations. A legend for each of the numbered settings is available in Table 2. The five patients are denoted by separate colored symbols, while bars represent the average values. It is generally assumed that the greater the NMI value, the superior the registration. A consistency exists in that registration settings, whether they perform poorly, admirably, or somewhere in between, perform similarly for all thoracic CT patients investigated.

Mean and individual longitudinal thoracic CT post‐deformable registration normalized mutual information values for all 36 setting combinations are plotted in Fig. 5 (see also legend in Table 2). Values suggest that the optimal setting for this particular application is stiff‐slow‐fine (Stiffness‐Speed‐Refinement); however, many other setting combinations yield very similar normalized mutual information values. For all five patient image pairs, three setting combinations consistently yield significantly lower NMI values than the other 33 settings and certainly should be avoided when registering thoracic CT images. Corresponding symmetric correlation ratio and correlation coefficient values demonstrate similar trends, suggesting that either metric can be used for ranking of registration settings.

**Table 2:** Legend to denote the various deformable registration setting combinations on Reveal‐MVS that are used in the Figures and text. The first two letters are used to abbreviate each of the user defined settings for: Stiffness (None, Soft, Medium, Stiff), Speed (Slow, Medium, Fast) and Refinement (Coarse, Medium, Fine).

*Combination*	*1*	*2*	*3*	*4*	*5*	*6*	*7*	*8*	*9*	*10*	*11*	*12*
Stiffness	Me	Me	Me	Me	Me	Me	Me	Me	Me	No	No	No
Speed	Fa	Fa	Fa	Me	Me	Me	Sl	Sl	Sl	Fa	Fa	Fa
Refinement	Co	Fi	Me	Co	Fi	Me	Co	Fi	Me	Co	Fi	Me
*Combination*	*13*	*14*	*15*	*16*	*17*	*18*	*19*	*20*	*21*	*22*	*23*	*24*
Stiffness	No	No	No	No	No	No	So	So	So	So	So	So
Speed	Me	Me	Me	Sl	Sl	Sl	Fa	Fa	Fa	Me	Me	Me
Refinement	Co	Fi	Me	Co	Fi	Me	Co	Fi	Me	Co	Fi	Me
*Combination*	*25*	*26*	*27*	*28*	*29*	*30*	*31*	*32*	*33*	*34*	*35*	*36*
Stiffness	So	So	So	St	St	St	St	St	St	St	St	St
Speed	Sl	Sl	Sl	Fa	Fa	Fa	Me	Me	Me	Sl	Sl	Sl
Refinement	Co	Fi	Me	Co	Fi	Me	Co	Fi	Me	Co	Fi	Me

**Figure 6:**
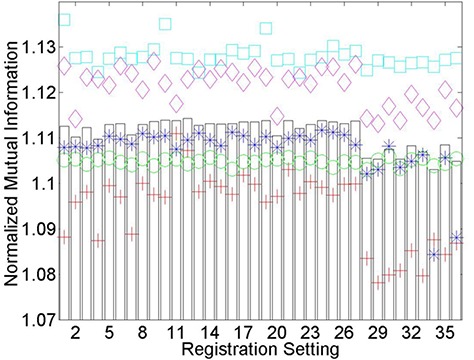
Normalized mutual information (NMI) values after the registration of pelvic MRI to planning CT images using each of the 36 registration settings on Reveal‐MVS. The five patients are denoted by separate colored symbols, while bars represent the average values.

An important observation from Fig. 5 is that registration settings, whether they perform poorly, admirably or somewhere in between, perform similarly for all thoracic CT patients investigated. This trend is also present for individual patient normalized mutual information values after the registration of pelvic MRI and CT images (Fig. 6), but perhaps not to the same degree. Similarity measure based rankings for the final three deformable registration applications investigated are summarized in Tables 3–5. Application dependence is evident considering None‐Medium‐Fine worked well for longitudinal PET and PET/CT deformable registration, but performed poorly in the registration of pelvic MVCT to CT images. Visual inspection of registration qualitatively reveals that a relationship exists between optimized similarity measure values and registration accuracy. This will be quantitatively verified for pelvic MR to CT registrations in section (D).

**Table 3:** Rankings of deformable registration settings used for the five longitudinal PET registrations. Separate rankings are based on mean postregistration normalized mutual information (NMI), symmetric correlation ratio (SCR), and correlation coefficient (CC) values, and are given for two of the best and two of the worst settings.

	*NMI*	*SCR*	*CC*
None‐Medium‐Fine	1	1	2
None‐Slow‐Fine	3	2	1
Stiff‐Fast‐Coarse	34	35	35
Stiff‐Slow‐Coarse	36	36	36

**Table 4:** Rankings of deformable registration settings used for the five PET/CT registrations. Separate rankings are based on mean postregistration normalized mutual information (NMI) and symmetric correlation ratio (SCR) values, and are given for two of the best and two of the worst settings.

	*NMI*	*SCR*
Soft‐Slow‐Fine	1	1
None‐Medium‐Fine	2	3
Stiff‐Slow‐Fine	34	35
Stiff‐Slow‐Coarse	36	36

**Table 5:** Rankings of deformable registration settings used for the five male pelvic MVCT to planning CT registrations. Separate rankings are based on mean postregistration normalized mutual information (NMI), symmetric correlation ratio (SCR), and correlation coefficient (CC) values, and are given for two of the best and two of the worst settings.

	*NMI*	*SCR*	*CC*
Soft‐Slow‐Coarse	1	4	2
Medium‐Slow‐Coarse	2	2	4
Soft‐Medium‐Fine	28	33	35
None‐Medium‐Fine	29	32	36

### C. Intra‐modality registration

**Figure 7:**
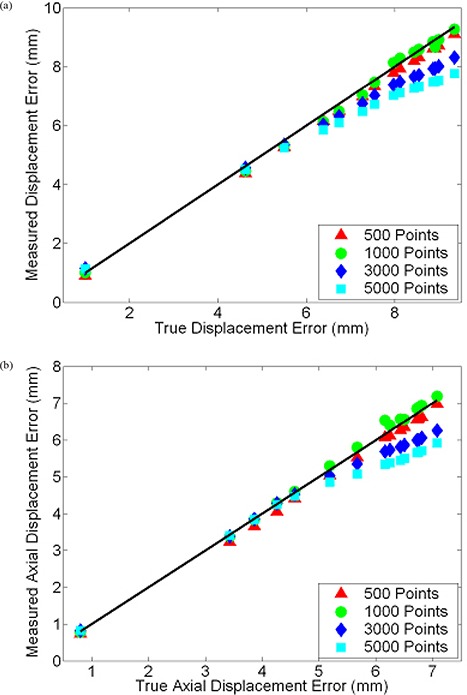
Plot (a) of displacement error (DE) values measured by our grid‐based method versus true DE values for various magnitudes of B‐spline warping. Either 500, 1000, 3000, or 5000 randomly located grid points were used. The solid dark line depicts correct measurement of true values. Similar plot (b) for axial components of DE.

**Figure 8:**
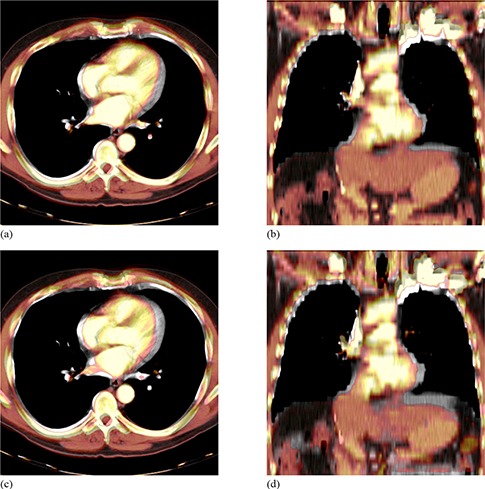
Sample colored warped images overlaid on greyscale baseline images depicting the range of magnitudes of synthetic B‐spline warped images used for validation of thoracic CT registrations: axial (a) and coronal (b) views of images that differ by a displacement error of 4.6 mm and an axial displacement error of 3.4 mm; axial (c) and coronal (d) views of images that differ by a displacement error of 9.4 mm and an axial displacement error of 7.1 mm.

Our method for determining approximate displacement error values for images registered on systems that do not provide access to the transformation function was tested by applying known B‐spline functions to a set of non‐uniform grid images. True displacement error and true axial displacement error values are plotted against the approximate values calculated by our in‐house software for each of the deformed grid images in Fig. 7. For reference, varying magnitudes of B‐spline warped images are depicted in Fig. 8. Measured and true displacement error values for all grid images show excellent agreement when true values are below 6 mm, at which faults in the measured values begin to increase for the grid images containing 3000 and 5000 points. This divergence of approximate values measured using our technique from true values also occurs when evaluating axial displacement errors greater than 5 mm. Presumably, for larger quantities of grid points and more complex deformations with greater displacement errors, the probability that the proximity of nearby grid points results in our software incorrectly determining corresponding grid points in original and deformed images increases. Considering this upper limit and the fact that statistics mandate a lower limit, we decided to use 1000 point grid images for intra‐modality registration assessment. Based on data points in Fig. 7, for deformations with displacement errors less than 9.35 mm and axial displacement errors less than 7.08 mm, on average our technique correctly measures these quantities within 0.13 mm and 0.11 mm, respectively. It requires mention that the reported accuracy of our grid based method of evaluating displacement errors is only applicable for the image matrix sizes, voxel dimensions and the registration application inherent to this particular section. Modification of any of these parameters may necessitate re‐assessment of the method.

**Figure 9:**
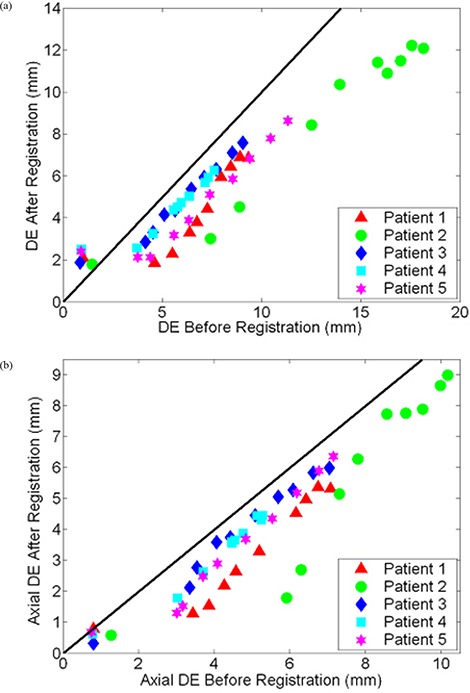
Scatter plot of post‐ versus predeformable registration (a) DE and (b) axial DE values for baseline and synthetically transformed thoracic CT images. Ten B‐spline warped images were registered to their respective original unwarped images on Reveal‐MVS for each of the five patients (denoted by separate colored symbols). A reference line with unity slope is shown in black.

After generating synthetic images by applying mathematically known deformations to patient images followed by the registration of synthetic to original thoracic CT images on Reveal‐MVS, postregistration axial and 3D displacement errors were evaluated. Resultant values are plotted against known predeformable registration values for all five patients in Fig. 9. If we assume the difference between pre‐ and postregistration DE values is a measure of improvement in image correspondence achieved by registration, correspondence increases for images that differ by initial displacement errors greater than approximately 4 mm. For initial DE values less than 2 mm, deformable registration on the commercial system appears to have negative effects.

### D. Inter‐modality registration

**Figure 10:**
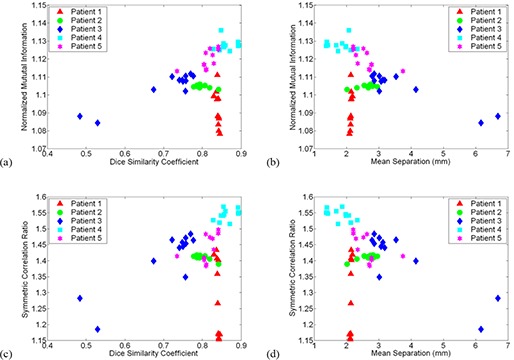
Relationship between image similarity measures and absolute prostate contour based metrics for 12 different settings in the registration of pelvic MRI to planning CT images: (a) normalized mutual information (NMI) versus Dice similarity coefficient (DSC); (b) NMI versus mean contour separation; (c) symmetric correlation ratio (SCR) versus DSC; (d) SCR versus mean separation. Each patient is represented by a different colored symbol.

Based on mean NMI and SCR values, the optimal registration settings for prostate MR to CT deformable registration are None‐Fast‐Medium and None‐Slow‐Medium, respectively. Pre‐ and postregistration prostate DSC, TC, and mean contour separation values for both settings are given in Table 6. Postregistration DSC and mean contour separation values were evaluated for ten additional setting combinations and were plotted against similarity measures in order to establish the relationship between relative and absolute validation metrics. Setting combinations were selected with the goal of including a spread of postregistration NMI and SCR values for each patient. NMI versus DSC, NMI versus mean separation, SCR versus DSC, and SCR versus mean separation are plotted in Fig. 10 for all five patients. For the most part, increased SCR and NMI similarity measure values correspond to improvements in the evaluated absolute validation metrics; however, this trend is clearly not universal. Results show that for one patient image pair and twelve setting combinations, a range of NMI and SCR values leads to little or no change in DSC or mean separation. Based on visual analysis of the original and deformed images, this apparent exception can be attributed to anatomical deviations between the original source and target images away from the prostate. This will be discussed in further detail in the following section.

**Table 6:** Prostate DSC, TC, and mean separation (MS) values for each of the five patients. Initial values are given for rigid alignment and after deformable registration of pelvic MRI and planning CT images, using two different settings on Reveal‐MVS.

		*Rigid*		*None‐Fast‐Medium*			*None‐Slow‐Medium*		
*P*	*DSC*	*TC*	*MS (mm)*	*DSC*	*TC*	*MS (mm)*	*DSC*	*TC*	*MS (mm)*
1	0.840	0.724	2.1	0.834	0.716	2.2	0.832	0.712	2.2
2	0.852	0.743	1.9	0.781	0.641	2.9	0.789	0.651	2.8
3	0.730	0.574	3.3	0.747	0.560	3.1	0.749	0.598	3.1
4	0.893	0.807	1.4	0.893	0.807	1.4	0.893	0.807	1.4
5	0.822	0.700	2.5	0.830	0.709	2.4	0.833	0.713	2.4

P=patient number

## IV. DISCUSSION

The use of phantoms in deformable registration validation is an ongoing area of discussion. Researchers continue to utilize them even though any phantom, no matter how complex the design, will not be able to simulate the range of anatomical variations that may occur in clinical imaging studies. We included a phantom study in our validation protocol not for quantified assessment, but with the intent that it may provide important insight on the tendencies of a black box commercial deformable image registration system. Based on phantom images, we established that the deformable registration algorithm on Reveal‐MVS must have strict constraints on the amount of local deformation that may occur. In addition, various intra‐ and inter‐modality phantom registrations revealed little or no evidence that the commercial system's apparent preference for global over local deformation is modality dependent. Although not quantifiable, these observations provide useful information that may be extrapolated to predict the system's ability to perform clinical deformable image registration applications. For example, if many local image deformations are required, this system may not be up for the task.

After establishing the optimal registration settings for five deformable registration applications through relative measures, absolute quantitative evaluation of an intra‐ and inter‐modality registration was performed. Intra‐modality deformable registration validation using known transformations is common in inter‐subject brain studies,^(^ ^26^ ^,^ ^41^ ^,^ ^42^ ^,^ ^43^ ^,^ ^44^ ^)^ but has had limited use in applications directly relevant to radiation therapy. By registering multiple B‐spline warped images to original thoracic CT images, we quantified the commercial system's performance over a range of potential initial clinical deformations. The resultant plot of postregistration versus preregistration displacement errors in Fig. 9 demonstrates a dual component trend in Reveal‐MVS's abilities. For the largest and progressively smaller initial DE values, correspondence is generally improved by deformable registration until a point is reached where postregistration DE values level off. Perhaps such plots or, more specifically, the location of the pivot point can be used as a standard for absolute comparison of deformable registration methods. Of course, further research is required to determine if the trend observed for Reveal‐MVS is consistent with other deformable registration algorithms.

The use of deformable registration instead of conventional rigid registration to improve structure delineation in the planning of prostate radiation therapy is an interesting concept. The predominant application of deformable registration of prostate MRI to CT images in the literature is to account for the deformation of the prostate caused by the insertion of an endorectal coil during magnetic resonance spectroscopic imaging (MRSI).^(^ ^21^ ^,^ ^30^ ^)^ Acquisition of anatomical MRI images with surface or other noninvasive coils significantly reduces problems related to prostate deformation but daily prostate motion^(^ ^45^ ^,^ ^46^ ^)^ remains a major concern. As a result, any translational offset between prostate positions in the MRI and CT with respect to surrounding anatomy may lead to inaccurate overlap of the MRI and CT prostate volumes after rigid registration. Based on our analysis in Table 6, Reveal‐MVS does not accurately account for internal prostate motion.

Although contour analysis was used for inter‐modality registration validation in this study, point landmark‐based evaluation is also an option. Both methods have the potential to provide useful quantitative information on the capabilities of a deformable registration system but, ultimately, we selected volumetric analysis simply because radiation therapy planning and delivery is based on doses delivered to volumetric regions of interest (ROI). However, for larger regions of interest that are susceptible to internal local deformations or ROI whose borders cannot be visually delineated with adequate accuracy, point‐based validation may be required. Ultimately, there may be some application dependence involvement in absolute inter‐modality deformable registration validation.

In additional to landmark‐based validation, two other validation methods excluded from our protocol have been the focus of recent research. The first is the consistency approach: in three given images (A, B, C), a comparison of transformations produced by registering A to B, B to C, and C to A provides a measure of registration error, assuming that errors are random and distributed evenly between each transformation.^(^ ^47^ ^)^ For the most part, consistency methods have been used for rigid registration validation,^(^ ^10^ ^)^ but have recently been applied to deformable models by Malsch et al.^(^ ^48^ ^)^ A novel registration assessment tool recently introduced is the concept of unbalanced energy^(^ ^49^ ^)^ whereby, instead of using gold standards, the physical fidelity of the deformation field is quantified through finite element models (FEMs). It has been applied with success to deformable registration of truncated pelvic CT images that include only a small region surrounding the prostate gland. Although promising, its relevancy for full 3D images of sites prone to significant anatomical deformations must be established. Like consistency testing, unbalanced energy certainly requires further research. Both validation methods were excluded from our protocol mainly because they rely heavily on the extensive knowledge of the deformation field which, as demonstrated by Reveal‐MVS, may not necessarily be the case for commercial systems.

The primary intent of this work was the development of a protocol for the assessment of commercial deformable registration systems, but while applying our protocol, two additional matters were also investigated. First, we developed and validated a method to compare true displacement fields with those produced by registration when the registration software does not allow access to the deformable transformation. We also tested the generally accepted notion that the quality of different deformable registration procedures can be ranked by comparing postregistration similarity measures. Whether the results of both investigations are specific to the deformable registration applications analyzed or are relevant to other applications requires further investigation.

In the known transformation study, we showed that by applying known B‐splines to a blank image with one thousand randomly positioned grid points followed by locating the discernible deformed grid points, the calculated displacement error from the sample points is highly accurate for a range of displacement error values. However, an upper limit is eventually reached due to our analysis software's reduced ability to correctly identify corresponding grid points in heavily deformed images. When registering images on the commercial system that differ by displacement error values greater than these upper limits, the accuracy of measured postregistration values may be questionable. Although an acceptable value for postregistration displacement errors has yet to be discussed, these upper limits certainly exceed any ideal acceptable quantity.

In using our random grid point method for DE evaluation of deformable registration on the Reveal‐MVS system, we make two fundamental assumptions. First, we assume that the commercial system improves or, at the very least, does not significantly reduce correspondence between the synthetically deformed and original thoracic CT images during the deformable registration procedure. The concern is that deformable registration may reduce correspondence to the point that postregistration displacement error values exceed the aforementioned threshold in which results can no longer be considered accurate and measured DE values may provide an erroneous picture of the system's capabilities. However, a problem such as this would be observable through visual inspection of registrations and this was certainly not the case. The second assumption is based on the accuracy of using a sample of voxels instead of the entire image to calculate displacement errors. We have demonstrated it to be acceptable for B‐splines, but whether or not the same is true for deformable transformations on the Reveal‐MVS system cannot be verified. Here the concern is that the commercial system performs significant local deformations whose contributions to the postregistration displacement error would not be picked up by sampling a limited number of voxels. Based on the observed global tendencies of Reveal‐MVS in comparison to B‐spline deformation, once again, we feel this is not a significant problem. In fact, part of the reason we chose to model synthetic transformations with B‐splines is that they are well‐known for allowing tremendous local control of image warping in comparison to other deformable models.^(^ ^50^ ^)^

**Figure 11:**
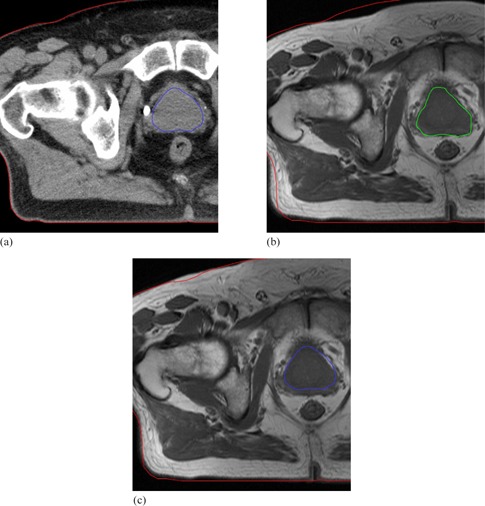
Axial slices of prostate patient whose MRI and CT images were acquired on different shaped couches: (a) CT with both the prostate and patient body outlined; (b) MRI after rigid alignment showing the MRI prostate and the CT patient contours (anatomical variation caused by the different couches is present in the bottom left hand corner); (c) CT prostate and patient contours overlaid on the MRI after deformable registration. Warping on Reveal‐MVS accounted for couch based anatomical variations but completely ignored the prostate motion.

The second investigation beyond the scope of the validation protocol was the establishment of a relationship between absolute measures of registration accuracy and post‐registration similarity measure comparison. Fig. 10 demonstrates that an increased SCR or NMI may not necessarily correspond to superior deformable registration of prostate MRI and CT images. For one patient in particular, registrations resulting in significantly different similarity measures led to little change in absolute contour based metrics. Further exploration revealed the source of the anomaly. All five CT images and all but one MRI images were acquired with the patient positioned on a flat imaging couch; however, the patient in question was scanned with a curved couch on the MRI unit. As a result, in addition to visible prostate motion, anatomical variations near the patient's posterior were present after rigid registration of the MRI and CT images. Deformable registration on Reveal‐MVS focused on patient deformation attributed to the different couches and essentially ignored the clearly visible prostate motion that had occurred between imaging studies. This is demonstrated for the optimal NMI registration setting in Fig. 11. In deforming the patient's backside, overall image similarity improved, but correspondence in the region of interest was compromised. Interestingly, an argument could be made that this result was predictable, based on qualitative observations in the phantom study. Regardless, anatomical variations away from clinically important sites must be factored into the practice of comparing deformable registrations based on similarity measure evaluation.

## V. CONCLUSIONS

We have presented a protocol for the validation of automatic commercial deformable image registration systems. Through a series of tests, the protocol provides a well‐balanced general assessment of a commercial system's capabilities. It initially consists of a qualitative phantom study to determine the system's general tendencies, followed by a relative comparison of the system's registration settings through similarity measure evaluation. The protocol also includes an assessment of the system's absolute intra‐modality registration accuracy using synthetic transformations and, finally, a contour‐based evaluation of the system's inter‐modality registration capabilities. Developed software and acquired images are readily available for assessment of other commercial systems that may be purchased for clinical or research objectives by our center. Access to the registration displacement field is not required as long as it can be stored and later re‐applied, which would probably be considered mandatory in a system approved for clinical use. The protocol is by no means static or definitive and can readily be expanded to investigate other potential deformable registration applications. Future work will include applying the protocol to additional deformable registration systems and the establishment of standard minimum benchmarks for the evaluated absolute measures.

## References

[1] Foskey M , Davis B , Goyal L , et al. Large deformation three‐dimensional image registration in image‐guided radiation therapy. Phys Med Biol. 2005;50(24):5869–92. 16333161 10.1088/0031-9155/50/24/008

[2] Gao S , Zhang L , Wang H , et al. A deformable image registration method to handle distended rectums in prostate cancer radiotherapy. Med Phys. 2006;33(9):3304–12. 17022225 10.1118/1.2222077

[3] Rietzel E , Chen GTY . Deformable registration of 4D computed tomography data. Med Phys. 2006;33(11):4423–30. 17153421 10.1118/1.2361077

[4] Pevsner A , Davis B , Joshi S , et al. Evaluation of an automated deformable image matching method for quantifying lung motion in respiration‐correlated CT images. Med Phys. 2006;33(2):369–76. 16532942 10.1118/1.2161408

[5] Heath E , Collins DL , Keall PJ , Dong L , Seuntjens J . Quantification of accuracy of the automated nonlinear image matching and anatomical labeling (ANIMAL) nonlinear registration algorithm for 4D CT images of lung. Med Phys. 2007;34(11):4409–21. 18072506 10.1118/1.2795824

[6] Boldea V , Sharp GC , Jiang SB , Sarrut D . 4D‐CT lung motion estimation with deformable registration: quantification of motion nonlinearity and hysteresis. Med Phys. 2008;35(3):1008–18. 18404936 10.1118/1.2839103

[7] Wu Z , Rietzel E , Boldea V , Sarrut D , Sharp GC . Evaluation of deformable registration of patient lung 4DCT with subanatomical region segmentations. Med Phys. 2008;35(2):775–81. 18383700 10.1118/1.2828378

[8] Schaly B , Kempe JA , Bauman GS , Battista JJ , Van Dyk J . Tracking the dose distribution in radiation therapy by accounting for variable anatomy. Phys Med Biol. 2004;49(5):791–805. 15070203 10.1088/0031-9155/49/5/010

[9] Lu W , Olivera G H , Chen Q , et al. Deformable registration of the planning image (kVCT) and the daily images (MVCT) for adaptive radiation therapy. Phys Med Biol. 2006;51(17):4357–74. 16912386 10.1088/0031-9155/51/17/015

[10] Hutton BF , Braun M . Software for image registration: algorithms, accuracy, efficacy. Semin Nucl Med. 2003:33(3):180–92. 12931320 10.1053/snuc.2003.127309

[11] Rohlfing T , Maurer CR , O'Dell WG , Zhong J . Modeling liver motion and deformation during the respiratory cycle using intensity‐based nonrigid registration of gated MR images. Med Phys. 2004;31(3):427–32. 15070239 10.1118/1.1644513

[12] Wu X , Dibiase SJ , Gullapalli R , Yu CX . Deformable image registration for the use of magnetic resonance spectroscopy in prostate treatment planning. Int J Radiat Oncol Biol Phys. 2004;58(5):1577–83. 15050339 10.1016/j.ijrobp.2003.09.072

[13] Rueckert D , Sonoda LI , Hayes C , Hill DLG , Leach MO , Hawkes DJ . Nonrigid registration using free‐form deformations: application to breast MR images. IEEE Trans Med Imaging. 1999;18(8):712–21. 10534053 10.1109/42.796284

[14] Wang H , Dong L , O'Daniel J , et al. Validation of an accelerated ‘demons’ algorithm for deformable image registration in radiation therapy. Phys Med Biol. 2005;50(12):2887–905. 15930609 10.1088/0031-9155/50/12/011

[15] Lu W , Chen M , Olivera GH , Ruchala KJ , Mackie TR . Fast free‐form deformable registration via calculus of variations. Phys Med Biol. 2004;49(14):3067–87. 15357182 10.1088/0031-9155/49/14/003

[16] Castadot P , Lee JA , Parrage A , Geets X , Macq B , Grégoire V . Comparison of 12 deformable registration strategies in adaptive radiation therapy for the treatment of head and neck tumors. Radiother Oncol. 2008;89(1):1–12. 18501456 10.1016/j.radonc.2008.04.010

[17] Pekar V , Gladilin E , Rohr K . An adaptive irregular grid approach for 3D deformable image registration. Phys Med Biol. 2006;51(2):361–77. 16394344 10.1088/0031-9155/51/2/012

[18] Guerrero T , Zhang G , Huang TC , Lin KP . Intrathoracic tumour motion estimation from CT imaging using the 3D optical flow method. Phys Med Biol. 2004;49(17):4147–61. 15470929 10.1088/0031-9155/49/17/022

[19] Kashani R , Hub M , Kessler ML , Balter JM . Technical note: a physical phantom for assessment of accuracy of deformable alignment algorithms. Med Phys. 2007;34(7):2785–88. 17821985 10.1118/1.2739812

[20] Serban M , Heath E , Stroian G , Collins DL , Seuntjens J . A deformable phantom for 4D radiotherapy verification: design and image registration evaluation. Med Phys. 2008;35(3):1094–102. 18404944 10.1118/1.2836417

[21] Lian J , Xing L , Hunjan S , et al. Mapping of the prostate in endorectal coil‐based MRI/MRSI and CT: a deformable registration and validation study. Med Phys. 2004;31(11):3087–94. 15587662 10.1118/1.1806292

[22] Crouch JR , Pizer SM , Chaney EL , Hu YC , Mageras GS , Zaider M . Automated finite‐element analysis for deformable registration of prostate images. IEEE Trans Med Imaging. 2007;26(10):1379–90. 17948728 10.1109/TMI.2007.898810

[23] Xiong L , Viswanathan A , Stewart AJ , et al. Deformable structure registration of bladder through surface mapping. Med Phys. 2006;33(6):1848–56. 16872092 10.1118/1.2198192

[24] Slomka PJ . Software approach to merging molecular with anatomic information. J Nucl Med. 2004;45 Suppl 1:36S–45S. 14736834

[25] Crum WR , Camara O , Hill DLG . Generalized overlap measures for evaluation and validation in medical image analysis. IEEE Trans Med Imaging. 2006;25(11):1451–61. 17117774 10.1109/TMI.2006.880587

[26] Lau YH , Braun M , Hutton BF . Non‐rigid image registration using a median‐filtered coarse‐to‐fine displacement field and a symmetric correlation ratio. Phys Med Biol. 2001;46(4):1297–319. 11324966 10.1088/0031-9155/46/4/326

[27] Bookstein F . Principal warps: thin plate splines and the decomposition of deformations. IEEE Trans Pattern Anal Mach Intell. 1989;11(6):567–85.

[28] Brock KK , Sharpe MB , Dawson LA , Kim SM , Jaffray DA . Accuracy of finite element model‐based multi‐organ deformable image registration. Med Phys. 2005;32(6):1647–59. 10.1118/1.1915012 16013724

[29] Alterovitz R , Goldberg K , Pouliot J , et al. Registration of MR prostate images with biomechanical modeling and nonlinear parameter estimation. Med Phys. 2006;33(2):446–54. 16532952 10.1118/1.2163391

[30] Venugopal N , McCurdy B , Hnatov A , Dubey A . A feasibility study to investigate the use of thin‐plate splines to account for prostate deformation. Phys Med Biol. 2005;50(12):2871–85. 15930608 10.1088/0031-9155/50/12/010

[31] Bharatha A , Hirose M , Hata N , et al. Evaluation of three‐dimensional finite element‐based deformable registration of pre‐ and intraoperative prostate imaging. Med Phys. 2001;28(12):2551–60. 11797960 10.1118/1.1414009

[32] Hayton P , Brady M , Tarassenko L , Moore N . Analysis of dynamic MR breast images using a model of contrast enhancement. Med Imag Anal. 1997;1(3):207–24. 10.1016/s1361-8415(97)85011-6 9873907

[33] Marais P , Brady JM . Detecting the brain surface in sparse MRI using boundary models. Med Imag Anal. 2000;4(3):283–302. 10.1016/s1361-8415(00)00020-7 11145314

[34] Vanuytsel LJ , Vansteenkiste JF , Stroobants SG , et al. The impact of (18)F‐fluoro‐2‐deoxy‐D‐glucose positron emission tomography (FDG‐PET) lymph node staging on the radiation treatment volumes in patients with non‐small cell lung cancer. Radiother Oncol. 2000;55(3):317–24. 10869746 10.1016/s0167-8140(00)00138-9

[35] Mac Manus MP , Hicks RJ , Ball DL , et al. F‐18 fluorodeoxyglucose positron emission tomography staging in radical radiotherapy candidates with nonsmall cell lung carcinoma: powerful correlation with survival and high impact on treatment. Cancer. 2001;92(4):886–95. 11550162 10.1002/1097-0142(20010815)92:4<886::aid-cncr1397>3.0.co;2-v

[36] Yap JT , Carney JPJ , Hall NC , Townsend DW . Image‐guided cancer therapy using PET/CT. Cancer J. 2004;10(4):221–33. 15383203 10.1097/00130404-200407000-00003

[37] Kagawa K , Lee WR , Schultheiss TE , Hunt MA , Shaer AH , Hanks GE . Initial clinical assessment of CT‐MRI image fusion software in localization of the prostate for 3D conformal radiation therapy. Int J Radiat Oncol Biol Phys. 1997;38(2):319–25. 9226318 10.1016/s0360-3016(96)00620-7

[38] Langen KM , Zhang Y , Andrews RD , et al. Initial experience with megavoltage (MV) CT guidance for daily prostate alignments. Int J Radiat Oncol Biol Phys. 2005;62(5):1517–24. 16029814 10.1016/j.ijrobp.2005.02.047

[39] Langen KM , Meeks SL , Poole DO , et al. The use of megavoltage CT (MVCT) images for dose recomputations. Phys Med Biol. 2005;50(18):4259–76. 16148392 10.1088/0031-9155/50/18/002

[40] Rasch C , Barillot I , Remeijer P , Touw A , van Herk M , Lebesque JV . Definition of the prostate in CT and MRI: a multi‐observer study. Int J Radiat Oncol Biol Phys. 1999;43(1):57–66. 9989514 10.1016/s0360-3016(98)00351-4

[41] D'Agostino ED , Maes F , Vandermeulen D , Seutens P . A viscous fluid model for multimodal non‐rigid image registration using mutual information. Med Image Anal. 2003;7(4):565–75. 14561559 10.1016/s1361-8415(03)00039-2

[42] Kybic J , Thévanez P , Nirkko A , Unser M . Unwarping of unidirectionally distorted EPI images. IEEE Trans Med Imaging. 2000;19(2):80–93. 10784280 10.1109/42.836368

[43] Xue Z , Shen D , Karacali B , Stern J , Rottenberg D , Davatzikos C . Simulating deformations of MR brain images for validation of atlas‐based segmentation and registration algorithms. Neuroimage. 2006;33(3):855–66. 16997578 10.1016/j.neuroimage.2006.08.007 PMC1752202

[44] Schnabel JA , Tanner C , Castellano‐Smith AD , et al. Validation of nonrigid image registration using finite‐element methods: application to breast MR images. IEEE Trans Med Imaging. 2003;22(2):238–47. 12716000 10.1109/TMI.2002.808367

[45] Langen KM , Jones DTL . Organ motion and its management. Int J Radiat Oncol Biol Phys. 2001;50(1):265–78. 11316572 10.1016/s0360-3016(01)01453-5

[46] Byrne TE . A review of prostate motion with considerations for the treatment of prostate cancer. Med Dosim. 2005;30(3):155–61. 16112467 10.1016/j.meddos.2005.03.005

[47] Woods RP , Grafton ST , Holmes CJ , Cherry SR , Mazziotta JC . Automated image registration: I. Genereal methods and intrasubject, intramodality validation. J Comput Assist Tomogr. 1998;22(1):139–52. 9448779 10.1097/00004728-199801000-00027

[48] Malsch U , Thieke C , Huber PE , Bendl R . An enhanced block matching algorithm for fast elastic registration in adaptive radiotherapy. Phys Med Biol. 2006;51(19):4789–806. 16985271 10.1088/0031-9155/51/19/005

[49] Zhong H , Peters T , Siebers JV . FEM‐based evaluation of deformable image registration for radiation therapy. Phys Med Biol. 2007;52(16):4721–38. 17671331 10.1088/0031-9155/52/16/001

[50] Lee S , Wolberg G , Chwa K , Shin SY . Image metamorphosis with scattered feature constraints. IEEE Trans Vis Comp Graph. 1996;2(4):337–54.

